# Differential Expression of *CADM1* in Gastrointestinal Stromal Tumors of Different Sites and with Different Gene Abnormalities

**DOI:** 10.3389/pore.2021.602008

**Published:** 2021-04-19

**Authors:** Jiayin Yuan, Takako Kihara, Neinei Kimura, Yuka Hashikura, Mizuka Ohkouchi, Koji Isozaki, Tsuyoshi Takahashi, Toshirou Nishida, Akihiko Ito, Seiichi Hirota

**Affiliations:** ^1^ Department of Surgical Pathology, Hyogo College of Medicine, Nishinomiya, Japan; ^2^ Departtment of Gastroenterological Surgery, Osaka University Graduate School of Medicine, Suita, Japan; ^3^ Japan Community Healthcare Organization (JCHO) Osaka Hospital, Osaka, Japan; ^4^ Department of Pathology, Faculty of Medicine, Kindai University, Osaka, Japan

**Keywords:** gastrointestinal stromal tumor, GIST, CADM1, gastric GIST, duodenal/jejuno-ileal GIST, mutation type

## Abstract

Gastrointestinal stromal tumor (GIST), the most common mesenchymal tumor of the human gastrointestinal tract, differentiating toward the interstitial cell of Cajal (ICC), arises predominantly in the stomach and small intestine. Small intestinal GISTs appear to have worse prognosis than gastric GISTs. In a pilot study of a cDNA expression chip using several GISTs, we found that Cell Adhesion Molecule 1 (CADM1), which could contribute to tumor growth and infiltration, is expressed more strongly in small intestinal GISTs than gastric GISTs. In the present study, we examined *CADM1* expression in GISTs of different sites and with different gene abnormalities using a large number of gastric and small intestinal GISTs. First, immunoblotting confirmed significantly higher CADM1 expression in small intestinal GISTs with exon 11 c-*kit* mutation than gastric GISTs with exon 11 c-*kit* mutation. Real-time PCR also revealed that small intestinal GISTs with exon 11 c-*kit* mutation showed significantly higher *CADM1* mRNA than gastric GISTs with exon 11 c-*kit* mutation. Although most small intestinal GISTs showed high *CADM1* mRNA expression regardless of gene abnormality types, different *CADM1* expression was detected between gastric GISTs with c-*kit* mutation and those with *PDGFRA* mutation. Immunohistochemistry showed that many small intestinal GISTs were CADM1-positive but most gastric GISTs CADM1-negative or -indefinite. In the normal gastric and small intestinal walls, immunoreactivity of CADM1 was detected only in nerves, but neither in gastric ICCs nor small intestinal ICCs, indicating that the high *CADM1*expression in small intestinal GISTs might be acquired during tumorigenesis. Different *CADM1* expression between gastric and small intestinal GISTs might be related to different prognoses between them. Further functional experiments are needed to elucidate the role of CADM1 on GIST biology, and there is a possibility that targeting therapy against CADM1 has a preventive effect for tumor spreading in small intestinal GISTs.

## Introduction

Gastrointestinal stromal tumor (GIST) is the most common mesenchymal tumor of the human gastrointestinal tract, differentiating toward the interstitial cell of Cajal (ICC) [1]. Sporadic GISTs usually harbor gain-of-function mutations in either c-*kit* (80–85%) or *PDGFRA* (5–10%) [2]. Most of the c-*kit* mutations in GISTs are present at exon 11, but a minority at exon 8, 9, 13 or 17 [3]. Characteristically, most GISTs with exon 9 c-*kit* mutations are located in the small intestine [4]. On the other hand, nearly all GISTs with *PDGFRA* mutations occur in the stomach. Approximately 10% of GISTs, defined as c-*kit*/*PDGFRA* wild-type (WT) GISTs [5], which carry wild-type sequences in all hot spots of c-*kit* and *PDGFRA*, represent some groups with distinct molecular characteristics, including defects in SDH complex [6], mutations of *NF1* [7], or mutations of *BRAF* [8].

GISTs show a wide spectrum of biologic behavior ranging from benign to malignant, varying by tumor size, mitotic activity and anatomical site. GISTs arise predominantly in the stomach (60–70%) and small intestine (20–30%) [9]. Some studies have suggested that the anatomical site has a prognostic value independent of tumor size and mitotic counts [10, 11]. Small intestinal GISTs usually show a higher malignant potential than gastric GISTs [11–13]. Distinct transcription profiles related to the anatomical location in GISTs have been reported [14, 15]. Hierarchical clustering analysis of the transcripts shows that GISTs are roughly separated into two groups such as gastric GISTs and small intestinal GISTs, and the result may partially explain why GISTs of the small intestine show more aggressive behavior than those of the stomach, with similar size and mitotic rate.

Cell adhesion molecule 1 (CADM1) is a member of the immunoglobulin superfamily initially known as spermatogenic immunoglobulin superfamily (SgIGSF) and synaptic cell adhesion molecule (SynCAM) [16–18]. CADM1 mediates formation of synapses between neurons, and adhesion between mast cells and nerve [17, 19], fibroblast [20], extracellular matrix [21] and muscular cells [19, 20] either homophilically or heterophilically. Moreover, CADM1 plays a role in adhesion between spermatogonia and Sertoli cells [22], or islet cells [23]. CADM1 was also identified as a tumor suppressor of lung cancer 1 (TSLC1) [24]. Loss of *CADM1* expression due to methylation was frequently found in many kinds of epithelial cancer [25], such as gastric cancer [26], breast cancer [27, 28], and squamous cell carcinoma [29–31]. In contrast, *CADM1* is upregulated in adult T cell leukemia and acute myelocytic leukemia, contributing to the enhancement of cell-cell adhesion to the vascular endothelium, tumor growth and tissue infiltration [32, 33].

In a pilot study using a small number of GIST cases, we found that small intestinal GISTs showed stronger expression of *CADM1* mRNA than gastric GISTs (data not shown). Here we assessed the expression of CADM1 at the protein and mRNA levels using a large number of gastric and small intestinal GISTs to clarify whether higher *CADM1* expression in small intestinal GISTs than gastric GISTs is really common and whether the expression of *CADM1* is different in GISTs with different types of gene abnormalities. We also performed immunofluorescent staining of CADM1 on normal gastric and small intestinal ICCs to examine whether the distinct expression of *CADM1* in GISTs at different anatomical sites is related to that in ICCs at different anatomical sites.

## Materials and Methods

### Tissue Specimens

In total, 107 cases previously diagnosed as GIST by pathological examination including KIT immunohistochemistry and c-*kit*/*PDGFRA* gene analyses were chosen for the present study. They included 76 fresh frozen GIST tissues (Table 1) and the other 31 formalin-fixed paraffin-embedded (FFPE) GIST tissues. Seventy-six fresh frozen GIST tissues from August 1995 to August 2016 at Hyogo College of Medicine Hospital and Osaka University Medical Hospital were used for Western blotting (16 cases) and real-time PCR experiments (62 cases) (Two cases were overlapped). Of 76 fresh frozen GISTs, 36 (47.4%) were duodenal/jejuno-ileal ones and 40 (52.6%) were gastric ones (Table 1). Of 36 duodenal/jejuno-ileal GISTs, 21 (58.3%) had exon 11 c-*kit* mutation, 10 (27.8%) had exon 9 c-*kit* mutation, and 5 (13.9%) had *NF1* abnormality (Table 1). Of 40 gastric GISTs, on the other hand, 21 (52.5%) had exon 11 c-*kit* mutation, 5 (12.5%) had exon 17 c-*kit* mutation and 14 (35.0%) had exon 18 *PDGFRA* mutation (Table 1). Tumor size, mitotic rate and clinical outcome of them were also shown in Table 1. Thirty-one cases of FFPE GIST tissues including 15 jejuno-ileal ones and 16 gastric ones were from Hyogo College of Medicine Hospital between 2007 and 2018 were used for immunohistochemistry. For CADM1 cDNA sequencing, 10 duodenal/jejuno-ileal GISTs among ones served for real-time PCR were used.

**TABLE 1:** Clinicopathological Characteristics of gastric and duodenal/jejuno-ileal GISTs examined for real-time PCR and Western blotting.

Case No.	Mutation site	Mutation type	Size (cm)	MR	FP (y) & A, DD or DO	WB	rPCR
G1	c*-kit*, 11	Trp557Gly	3.5	0	2.1, A	+	−
G2	c*-kit*, 11	Val559Asp	4	0	3.5, A	+	−
G3	c*-kit*, 11	Val559Asp	2.6	2	3.4, A	+	−
G4	c*-kit*, 11	Val559Asp	6.1	4	3.5, A	+	−
G5	c-kit, 11	Val559Gly	3.3	1	3.6, A	+	−
G6	c*-kit*, 11	Val560Asp	3	2.5	3.5, A	+	−
G7	c*-kit*, 11	5a.a. (551-555) to Leu	2	6	5.0, A	+	−
G8	c*-kit*, 11	20a.a. (554-573) to Thr	1.9	6	5.3, A	+	−
G9	c*-kit*, 11	Trp557Arg	3	0	10.8, A	−	+
G10	c*-kit*, 11	Trp557Arg	2.5	0	4, A	−	+
G11	c*-kit*, 11	Val559Asp	6	0	13.3, A	−	+
G12	c*-kit*, 11	Leu576Pro	3	6	3.5, A	−	+
G13	c*-kit*, 11	Val560Glu	3.2	n.a.	4.3, A	−	+
G14	c*-kit*, 11	Del-Val560	6.5	5	3.4, A	−	+
G15	c*-kit*, 11	Del-Asp579	3.5	1	5.6, A	−	+
G16	c*-kit*, 11	Del-Asp579	3.5	2	1.6, A	−	+
G17	c*-kit*, 11	Del-Asp579	2.5	1	10, A	−	+
G18	c*-kit*, 11	Del-Asp579	4	3	4.8, A	−	+
G19	c*-kit*, 11	Del-9a.a. (550-558)	2	12	3.6, A	−	+
G20	c*-kit*, 11	Del-5a.a. (554-558)	2.6	140	0.9, A	−	+
G21	c*-kit*, 11	Dup-7a.a. (573-579)	3	1	8.2, A	−	+
G22	c*-kit*, 17	Asn822Lys	3.8	<5	8.8, A	−	+
G23	c*-kit*, 17	Asn822Lys	10	0	0.3, A	−	+
G24	c*-kit*, 17	Asn822Lys	2.5	1	n.a.	−	+
G25	c*-kit*, 17	Asn822Lys	5.3	2	1.6, A	−	+
G26	c*-kit*, 17	Asn822Lys	30	3	n.a.	−	+
G27	*PDGFRA*, 18	Asp842Val	4	2	13.5, A	−	+
G28	*PDGFRA*, 18	Asp842Val	2	2	7.2, A	−	+
G29	*PDGFRA*, 18	Asp842Val	14	3	5.1, A	−	+
G30	*PDGFRA*, 18	Asp842Val	14	0	10.3, A	−	+
G31	*PDGFRA*, 18	Asp842Val	3.5	0	5.1, A	−	+
G32	*PDGFRA*, 18	Asp842Val	n.a.	35	n.a.	−	+
G33	*PDGFRA*, 18	Asp842Val	4.5	0	5, A	−	+
G34	*PDGFRA*, 18	Asp842Val	7	3	8.1, A	−	+
G35	*PDGFRA*, 18	Asp842Val	2.8	0	1, A	−	+
G36	*PDGFRA*, 18	Asp842Val	4	n.a.	2, A	−	+
G37	*PDGFRA*, 18	Asp842Val	3	<5	3.8, A	−	+
G38	*PDGFRA*, 18	Asp842Val	3	n.a.	3.8, A	−	+
G39	*PDGFRA*, 18	Del-4a.a. (842-845)	3	1	5.7, A	−	+
G40	*PDGFRA*, 18	Del-4a.a. (843-846)	3.5	1	4.7, A	−	+
D1	c*-kit*, 9	Dup-2a.a. (502&503)	10	40	3.2, A	−	+
D2	c*-kit*, 9	Dup-2a.a. (502&503)	3.5	50	0.3, A	−	+
D3	c*-kit*, 9	Dup-2a.a. (502&503)	10.5	10	2.4, DD	−	+
D4	c*-kit*, 9	Dup-2a.a. (502&503)	3.5	<10	n.a.	−	+
D5	c*-kit*, 9	Dup-2a.a. (502&503)	11	2	n.a.	−	+
D6	c*-kit*, 11	Del-2a.a. (557&558)	11	15	n.a.	−	+
D7	c*-kit*, 11	Val559Ala	5	1	n.a.	−	+
D8	c*-kit*, 11	Val560Asp	8	4	6.1, A	−	+
D9	c*-kit*, 11	Leu576Pro	10	0	3.4, A	−	+
D10	c*-kit*, 11	Leu576Pro	10	0	3.4, A	−	+
D11	c*-kit*, 11	8a.a. (559-566) to Asp	n.a.	n.a.	n.a.	−	+
S1	c*-kit*, 9	Dup-2a.a. (502&503)	3.8	40	5.3, A	−	+
S2	c*-kit*, 9	Dupli-2a.a. (502&503)	3.5	5	n.a.	−	+
S3	c*-kit*, 9	Dup-2a.a. (502&503)	20	24	7, A	−	+
S4	c*-kit*, 9	Dup-2a.a. (502&503)	3.5	<5	5.1, A	−	+
S5	c*-kit*, 9	Dup-2a.a. (502&503)	4.2	8	6.6, A	−	+
S6	c*-kit*, 11	Del-6a.a. (554-559)	16	2	3.5, A	+	−
S7	c*-kit*, 11	Del-2a.a. (555&556)	10	1	3.5, A	+	−
S8	c*-kit*, 11	Trp557Gly	3	0	3.6, A	+	−
S9	c*-kit*, 11	Trp557Gly	8	<5	5.1, A	+	−
S10	c*-kit*, 11	Val559Ala	6	30	5.7, A	+	−
S11	c*-kit*, 11	Val560Gly	8.2	15	5.0, A	+	−
S12	c*-kit*, 11	9a.a. (565-573) to Tyr/Val/Ser	15	4	4.8, A	+	+
S13	c*-kit*, 11	Dup-5a.a. (580-584)	4.2	3	7.7, A	+	+
S14	c*-kit*, 11	Trp557Gly	5	150	0.9, DD	−	+
S15	c*-kit*, 11	Val559Ala	11	1	6.4, A	−	+
S16	c*-kit*, 11	Leu576Pro	2.5	0	n.a.	−	+
S17	c*-kit*, 11	Del-Leu576	11	50	8.8, A	−	+
S18	c*-kit*, 11	Del-3a.a. (577-579)	11	n.a.	5.8, A	−	+
S19	c*-kit*, 11	3a.a. (552-554) to Lys	5.5	1	2.3, A	−	+
S20	c*-kit*, 11	4a.a. (556-559) to His	10	1	15.4, A	−	+
S21	*NF-1*	NF-1	5.5	1	4.3, A	−	+
S22	*NF-1*	NF-1	4.5	1	3.9, a	−	+
S23	*NF-1*	NF-1	3	5	n.a	−	+
S24	*NF-1*	NF-1	2	<5	5.1, DO	−	+
S25	*NF-1*	NF-1	0.5	0	n.a	−	+

G, D and S mean gastric GIST, duodenal GIST and small intestinal GIST other than duodenal GIST, respectively. Numbers in mutation site mean exon number of the mutation. Mitotic rate (MR) represents mitotic number per 50 high power fields. Follow-up period (FP) refers to the length of time from the date of surgery to patients' last visit. A, alive; DD, dead of GIST; DO, dead of other disease. rPCR, real-time PCR. WB, western blotting. n.a., data not available

### Western Blotting

Equals of protein samples extracted from fresh frozen tissues of eight small intestinal (jejuno-ileal) GISTs with exon 11 c-*kit* mutation and eight gastric GISTs with exon 11 c-*kit* mutation were separated by 12% SDS-PAGE and subsequently transferred to PVDF membrane (Invitrogen, Carlsbad, CA). Membrane was incubated with anti-CADM1 chicken monoclonal antibody (MBL International, Woburn, MA) diluted at 1:1,000 for 3 h at room temperature using iBind^TM^ Western System (Invitrogen). Immunoreactive protein bands were visualized using Prime Western Blotting Detection Reagent (GE Healthcare Life Science, Buckinghamshire, United Kingdom) on ChemiDoc Imaging System (Bio-Rad, Laboratories, California, United States). Reprobing by anti-KIT rabbit polyclonal antibody (DAKO, Carpinteria, CA) and anti-β-actin mouse monoclonal antibody (Abcam, Cambridge, United Kingdom) was done.

### Real-Time Quantitative RT-PCR

Total RNA was extracted from 62 fresh frozen GIST tissues using RNeasy Mini Kit (QIAGEN, Hilden, Germany) according to the manufacturer’s recommendation. Complementary DNA was synthesized from total RNA (10 μg) using reverse transcriptase (Invitrogen). Real-time PCR analysis was performed using the Applied Biosystems STEP ONE^TM^ standard real-time PCR system (Applied Biosystems, Foster City, CA) based on published sequences for genes encoding the human CADM1, c-*kit*, and GAPDH (Applied Biosystems). For each sample, total volume of 20 μl consisted of 10 μl TaqMan Gene Expression Master Mix (2x, Applied Biosystems), 1 μl TaqMan Probe, 1 μl of each cDNA template and 8 μl of distilled water. The real-time PCR fragments were amplified as follows: one cycle at 48°C for 30 min, 40 cycles at 95°C for 10 min and 60°C for 1 min. Expression level of *CADM1* mRNA and c-*kit* mRNA was standardized by expression of *GAPDH* mRNA.

### Immunohistochemistry

Three μm-thick sections were cut from formalin-fixed and paraffin embedded tissues, mounted on slides coated with 3-aminopropyltriethoxysilane, deparaffinized in xylene and rehydrated in descending grades of ethanol. Antigen retrieval was performed in a water bath for 20 min at 98°C at Target Retrieval Solution, pH 9.0 (DAKO). Nonspecific staining was blocked by treating the sections with 5% donkey serum in Tris-buffered saline (pH 6.0) for 30 min. CADM1 immunohistochemistry was performed with a monoclonal chicken antibody (3E1, MBL International) diluted at 1:1,000 in Antibody Diluent (DAKO). The slides were incubated with donkey anti-chicken IgY-HRP conjugated (EMD Millipore, Temecula, CA) as a secondary antibody at 1:500 dilution. Signals were developed with diaminobenzidine substrate chromogen system (DAKO), and counterstained with hematoxylin. Images were obtained under All-in-One Fluorescence Microscope (KEYENCE, Osaka, Japan).

### Immunofluorescence Staining

Deparaffinization and rehydration of the sections were performed as described. After the sections were autoclaved for 20 min at 121°C in Target Retrieval Solution, pH 9.0 (DAKO), they were doubly immunostained with a combination of the anti-CADM1 antibody (MBL International) diluted at 1:1,000 and anti-c-*kit* antibody (Novocastra^TM^) diluted at 1:100 for 1 h in room temperature, followed by Cy5-conjugated anti-chicken IgY and Cy2-conjugated anti-rabbit IgG secondary antibodies (Jackson ImmunoResearch, West Grove, PA), respectively in dark for 45 min. The slides then mounted with antifade mountant with DAPI (GE Healthcare Life Science), and examined with a laser confocal scanning system (ZEISS, LSM780, Oberkochen, Germany) under 510 and 670 nm.

### cDNA Sequencing of *CADM1*

Total RNA extracted from 10 fresh small intestinal GIST tissues with high CADM1 expression was reverse-transcribed to cDNA, and PCR for *CADM1* cDNA amplification was performed. Primer sequences are listed in Supplementary Table S1. PCR products were purified with the Qiaquick Gel Extraction Kit (QIAGEN) and sequenced on both strands using Big Dye Terminator v3.1 Cycle Sequencing kit (Applied Biosystems) and ABI 3100-Avant Genetic Analyzer (Applied Biosystems).

### Statistical Analysis

Statistical analyses were performed using Prism GraphPad 8 (GraphPad Software, La Jolla, CA). Two-tailed unpaired Student’s *t*-tests with Welch’s correction or one-way ANOVA for multiple comparisons when appropriate were used to determine statistical significance (*p* < 0.05 was considered statistically significant).

## Results

### Expression of CADM1 Protein in Gastric and Small Intestinal GISTs with Exon 11 c-*Kit* Mutation Examined by Western Blotting

**FIGURE 1:**
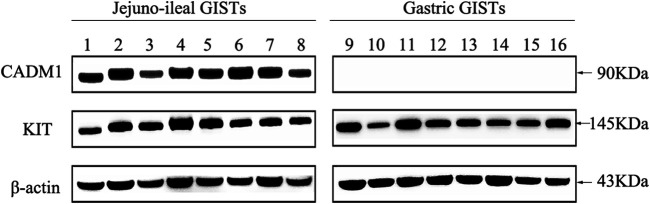
Distinct expression of CADM1 protein between small intestinal and gastric GISTs with exon 11 c-*kit* mutation by Western blotting. CADM1 protein was abundantly detected in all cases (n = 8) of small intestinal (jejuno-ileal) GISTs with exon 11 c-*kit* mutation, while that was not apparent in all gastric GISTs with exon 11 c-*kit* mutation (n = 8). All jejuno-ileal and gastric GISTs with exon 11 c-*kit* mutation examined strongly expressed KIT protein, and expression of β-actin as a control was normally detected in all samples.

CADM1 expression at the protein level was assessed in eight small intestinal (jejuno-ileal) and eight gastric GISTs with exon 11 c-*kit* mutation (Table 1) by Western blotting. Apparent bands of CADM1 protein were detected in all small intestinal GISTs with exon 11 c-*kit* mutation but not in all gastric GISTs with exon 11 c-*kit* mutation (Figure 1). All small intestinal and gastric GISTs with exon 11 c-*kit* mutation examined strongly expressed KIT protein (Figure 1).

### Expression of CADM1 mRNA in Gastric and Small Intestinal GISTs with Various Gene Abnormalities Examined by Real-Time PCR

**FIGURE 2:**
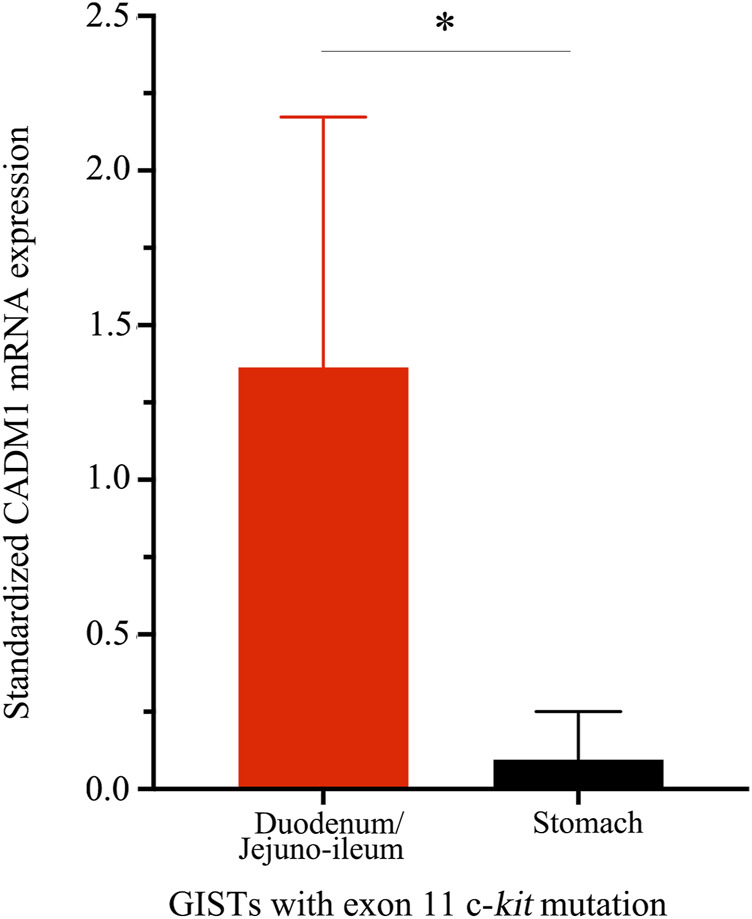
Distinct *CADM1* mRNA levels quantified by real-time PCR between small intestinal and gastric GISTs with exon 11 c-*kit* mutation. Quantitative RT-PCR demonstrated that level of *CADM1* mRNA expression standardized by *GAPDH* mRNA expression in small intestinal GISTs with exon 11 c-*kit* mutation (n = 15) and that in the gastric GISTs with exon 11 c-*kit* mutation (n = 13) was statistically significant (*p < 0.0001).

**FIGURE 3:**
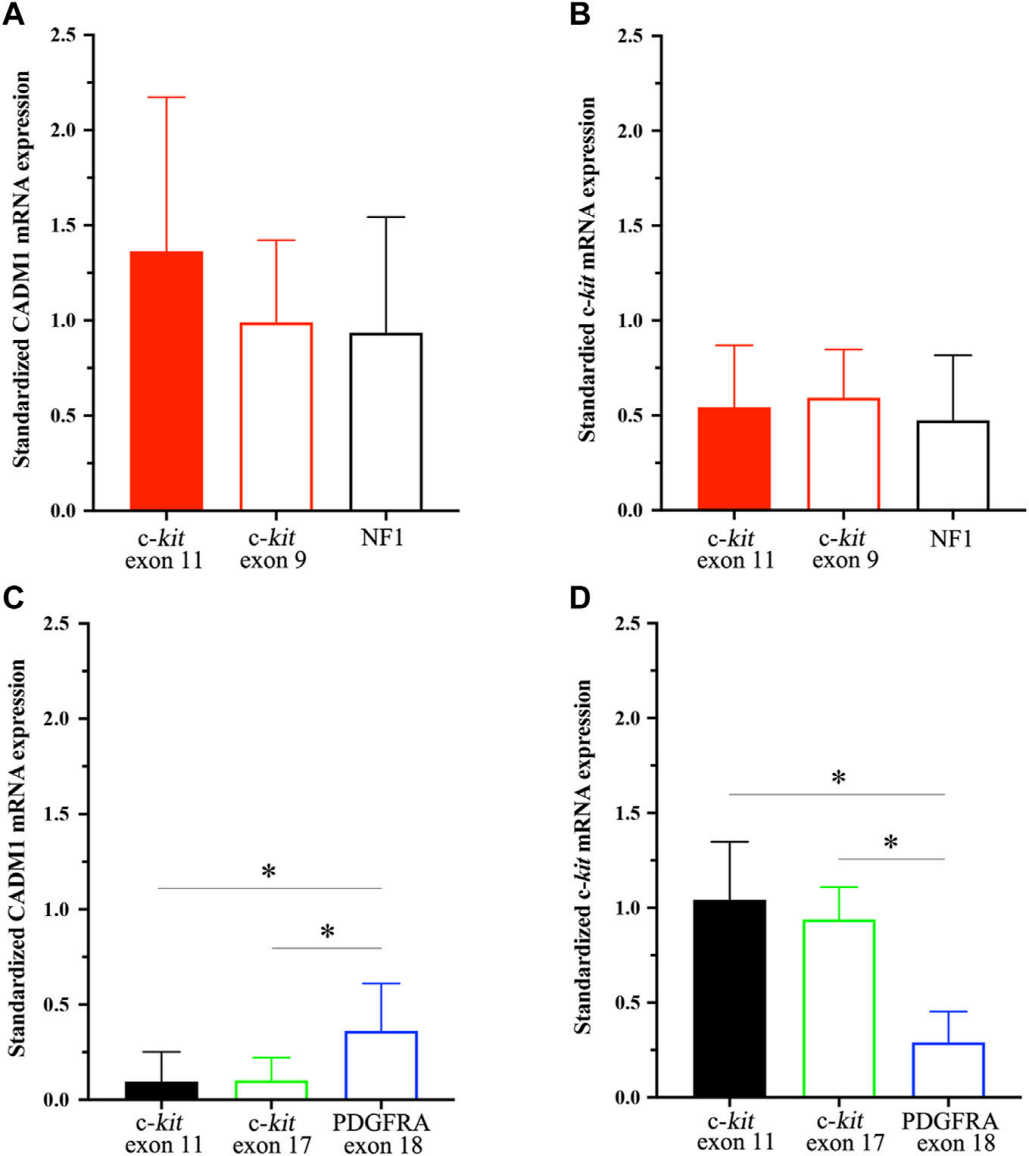
*CADM1* and c-*kit* mRNA levels quantified by real-time PCR in small intestinal GISTs and gastric GISTs with different gene abnormalities. **(A)**. *CADM1* mRNA expression level standardized by *GAPDH* mRNA expression showed no significant difference among small intestinal GISTs with exon 11 c-*kit* mutation, those with exon 9 c-*kit* mutation and those from NF1 patients. **(B)**. Expression level of c-*kit* mRNA standardized by *GAPDH* mRNA expression was similar among small intestinal GISTs with exon 11 c-*kit* mutation, those with exon 9 c-*kit* mutation and those from NF1 patients. **(C)**. *CADM1* mRNA expression level standardized by *GAPDH* mRNA expression in gastric GISTs with exon 18 *PDGFRA* mutation was significantly higher than that with exon 11 c-*kit* mutation (*p* = 0.0028) or exon 17 c-*kit* mutation (*p* = 0.0403). **(D)**. Expression level of c-*kit* mRNA standardized by *GAPDH* mRNA expression in gastric GISTs with exon 18 *PDGFRA* mutation was significantly lower than that with exon 11 or exon 17 c-*kit* mutation (*p* < 0.001). * means statistical significance (*p* < 0.05).

CADM1 mRNA expression was quantified by real-time PCR in gastric and small intestinal GISTs with various gene abnormalities. First, we compared expression level of CADM1 mRNA in duodenal/jejuno-ileal GISTs with exon 11 c-*kit* mutation and that in gastric GISTs with exon 11 c-*kit* mutation (Table1). Significantly higher expression of CADM1 mRNA was observed in 15 duodenal/jejuno-ileal GISTs with exon 11 c-*kit* mutation than in 13 gastric GISTs with exon 11 c-*kit* mutation (*p* < 0.0001, Figure 2), consistent with the protein analysis (Figure 1). Next, expression level of CADM1 mRNA was compared among duodenal/jejuno-ileal GISTs with various gene abnormalities (Table 1). Ten duodenal/jejuno-ileal GISTs with exon 9 c-*kit* mutation and five those from NF1 patients (Table 1) showed high expression of CADM1 mRNA, similar to those with exon 11 c-*kit* mutation (Figure 3A). All types of duodenal/jejuno-ileal GISTs with different gene abnormalities also showed similar levels of c-*kit* mRNA (Figure 3B). Then, we compared expression level of CADM1 mRNA among gastric GISTs with various gene abnormalities (Table 1). Fourteen gastric GISTs with exon 18 *PDGFRA* mutation expressed significantly higher mRNA of CADM1 than 13 those with exon 11 c-*kit* mutation (*p* = 0.0028) and five those with exon 17 c-*kit* mutation (*p* = 0.0403) (Table 1; Figure 3C), but the level of CADM1 mRNA expression even in gastric GISTs with exon 18 *PDGFRA* mutation was significantly lower than that in small intestinal GISTs with various types of gene abnormalities (*p* = 0.0005) (Figures 3A,C). Expression of c-*kit* mRNA in gastric GISTs with exon 11 and exon 17 c-*kit* mutations was significantly higher (*p* = 0.0004 and *p* = 0.0200, respectively) than that in small intestinal GISTs with exon 11 c-*kit* mutation (Figures 3B,D), but that in gastric GISTs with exon 18 *PDGFRA* mutation was significantly lower than that in gastric GISTs with exon 11 and exon 17 c-*kit* mutation (*p* < 0.0001 and *p* < 0.0001, respectively) (Figure 3D).

### Immunohistochemical and Immunofluorescent Staining of CADM1 in Gastric and Small Intestinal GISTs

**FIGURE 4:**
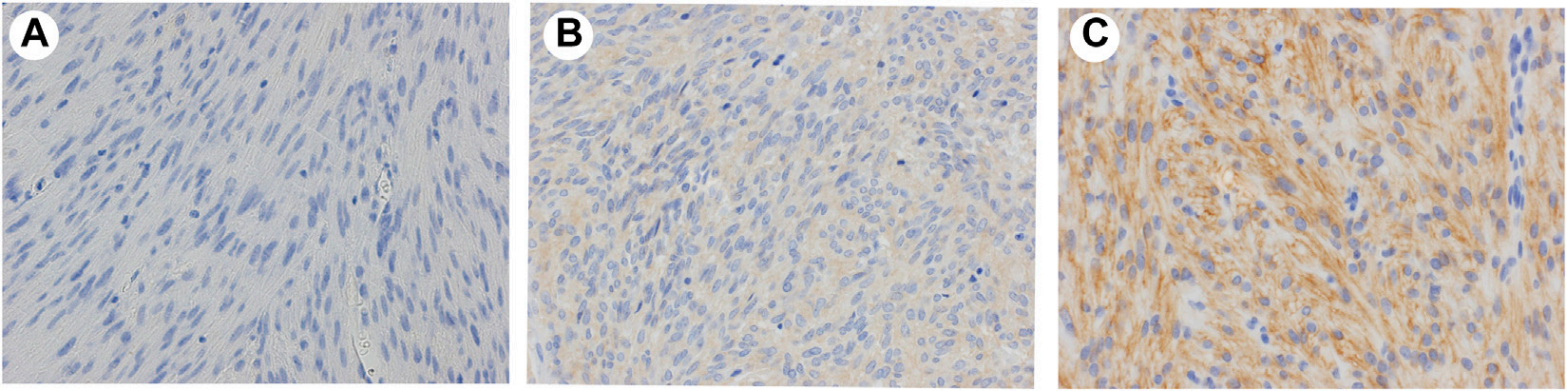
Representative results of expression of CADM1 immunostaining in GISTs. **(A)**. Negative: no apparent CADM1 staining. **(B)**. Indefinite: weak CADM1 cytoplasmic staining but no membranous staining. **(C)**. Positive: diffuse or focal strong CADM1 membranous staining. **(A)**. Gastric GIST. **(B)** and **(C)**. Small intestinal GISTs. Original magnification: ×400.

**FIGURE 5:**
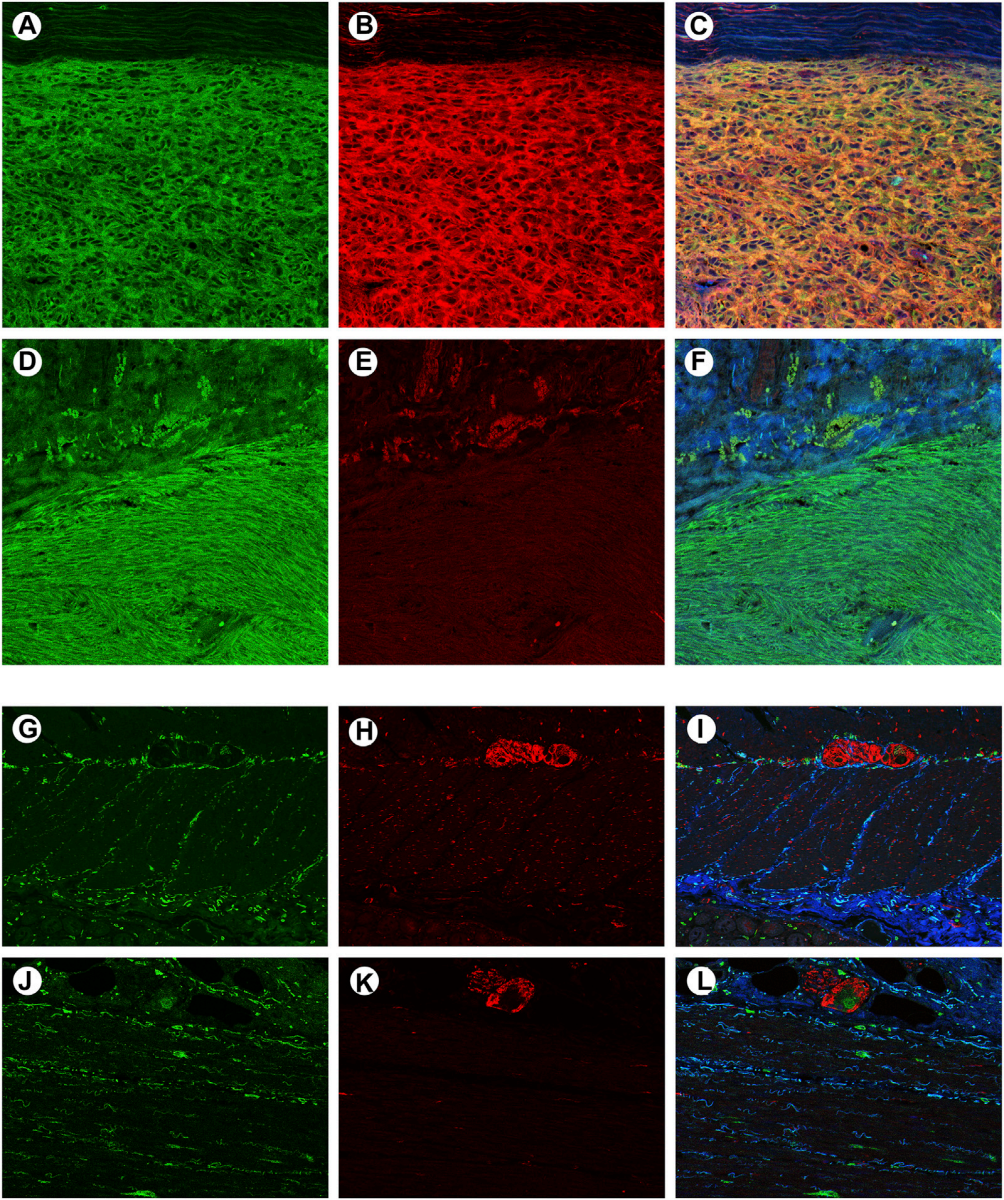
Confocal microscopic images of immunofluorescent staining with KIT and CADM1 in a representative small intestinal GIST, a representative gastric GIST, normal small intestinal wall and normal gastric wall. **(A)**. The small intestinal GIST was stained by KIT (*green*). **(B)**. The small intestinal GIST was also stained by CADM1 (*red*), **(C)**. Merged image of panels “a” and “b” showed *yellow* color, indicating co-expression of c-*kit* and CADM1 by the tumor tissue. **(D)**. The gastric GIST was stained with KIT (*green*). **(E)**. No apparent staining with CADM1 was detected in the gastric GIST. **(F)**. Merged image of panels “d” and “e” was showed only *green* color. **(G)**. KIT-positive cells (*green*) were located around the myenteric plexus and within the muscular layers in small intestinal wall. **(H)**. Immunoreactivity for CADM1 (*red*) was only observed in nerve strands. **(I)**. Merged image of KIT and CADM1 immunoreactivities showed no doubly stained cells in small intestinal wall. **(J)**. KIT-positive cells (*green*) were also detected around the myenteric plexus in gastric wall. **(K)**. CADM1-positive cells (*red*) were only seen in nerve tissues. l. The merged image of KIT and CADM1 immunoreactivities did not show double positive cells in gastric wall. a-l. Original magnification: ×200.

To confirm whether CADM1 protein is expressed by tumor cells themselves of small intestinal GISTs, immunohistochemistry for CADM1 was performed. According to membranous staining pattern, CADM1 expression was divided into three categories; no staining (negative) (Figure 4A), weak staining (indefinite) (Figure 4B) and strong staining (positive) (Figure 4C). Diffuse and strong immunoreactivity (positive) for anti-CADM1 was observed in 11 cases (73.3%) of small intestinal GISTs, but the others had no staining (negative) in 2 cases or weak staining (indefinite) in 2 cases. Only 1 of 16 (6.25%) gastric GISTs showed focal positive staining for CADM1, 9 no staining (negative), and six weak staining (indefinite). Immunofluorescent staining of CADM1 (Figure 5A) and KIT (Figure 5B) in small intestinal GISTs clearly showed double positivity of them (Figure 5C). On the other hand, gastric GISTs showed KIT expression (Figures 5E,F) but not CADM1 expression (Figures 5D,F).

### Immunofluorescence Staining of CADM1 in Normal Gastric and Small Intestinal Walls

To clarify the localization of CADM1 protein in normal gastrointestinal tract, double immunofluorescence staining of KIT and CADM1 was performed in normal small intestinal and gastric walls. In the small intestinal wall, KIT-positive ICCs were located around the myenteric plexus and in the muscular layers (Figure 5G). On the other hand, immunoreactivity for CADM1 was only observed at nerve strands (Figure 5H). Thus, the merged image of KIT immunoreactivity and CADM1 immunoreactivity showed no doubly stained cells (Figure 5I). In the wall of the stomach, KIT-positive ICCs were also observed around the myenteric plexus and in the muscular layers (Figure 5J). CADM1 immunofluorescence staining showed that the positive cells were only present at the myenteric plexus (Figure 5K). KIT immunoreactivity and CADM1 immunoreactivity were not overlapped (Figure 5L).

### Mutational Analysis of *CADM1* Gene in Small Intestinal GISTs

We investigated whether small intestinal GISTs with high expression of *CADM1* mRNA had the *CADM1* gene mutations. cDNA sequencing analysis of *CADM1* was performed in 10 duodenal/jejuno-ileal GISTs, resulted in no detection of *CADM1* gene alteration (data not shown).

## Discussion

CADM1 is an adhesion molecule which is implicated in tumor invasion of adult T-cell leukemia cells and acute myelocytic leukemia cells [32, 33]. Different prognoses between patients with gastric GISTs and those with small intestinal GISTs have been reported [11–13], and different protein expression between them is considered to be a possible explanation of the different prognoses. We noticed that *CADM1* might be highly expressed by small intestinal GISTs compared to gastric GISTs by cDNA expression chip using a small number of gastric and small intestinal GISTs. In the present study, we tried to clarify whether higher *CADM1* expression in small intestinal GISTs than gastric GISTs is really common in a large number of GIST cases, and also examined whether the expression of *CADM1* is similar or not in GISTs with different types of gene abnormalities.

First, we performed CADM1 immunoblotting using eight representative gastric GISTs with exon 11 c-*kit* mutation and eight representative small intestinal GISTs with exon 11 c-*kit* mutation. All of the small intestinal GISTs showed high expression of CADM1 protein but all of the gastric GISTs did not show apparent signal. The quantitative RT-PCR also revealed that the level of *CADM1* gene transcript was significantly different between gastric GISTs with exon 11 c-*kit* mutation and small intestinal GISTs with exon 11 c-*kit* mutation. We considered that gastric GISTs and small intestinal GISTs show different level of *CADM1* expression even in the GISTs with the same exon 11 c-*kit* mutation.

In small intestinal GISTs, those with different gene abnormalities such as those with exon 9 c-*kit* mutation and those in NF1 patients similarly expressed high level of *CADM1* mRNA as observed in those with exon 11 c-*kit* mutation. We concluded that *CADM1* is commonly upregulated in all types of small intestinal GISTs with different gene alterations. On the other hand, most of the gastric GISTs with exon 17 c-*kit* mutation showed extremely low expression of *CADM1* mRNA as observed in those with exon 11 c-*kit* mutation, but the gastric GISTs with exon 18 *PDGFRA* mutation expressed moderate level of *CADM1* mRNA compared with those with exon 11 c-*kit* mutation. Thus, gastric GISTs were considered to show different level of *CADM1* in those with different type of gene abnormalities. Interestingly, one gastric GIST with exon 9 c-*kit* mutation showed upregulation of *CADM1* mRNA (data not shown). Since most GISTs with exon 9 c-*kit* mutation are located in the small intestine, this gastric GIST with exon 9 c-*kit* mutation may belong to so-called ‘small intestinal subtype of GIST’ from viewpoints of c-*kit* mutation type and *CADM1* mRNA expression.

The different expression levels of *CADM1* mRNA between gastric and small intestinal GISTs suggested that the progenitor cells (i.e. ICCs) might have different expression pattern of *CADM1* in normal gastric and small intestinal walls. However, we could not detect CADM1 protein not only in gastric ICCs but also in small intestinal ICCs by double immunofluorescence staining of KIT and CADM1. Small intestinal GISTs might acquire *CADM1* expression during tumorigenesis from precursor of GISTs (i.e. ICCs) to GISTs.

In our experiment, some discrepancy concerning *CADM1* expression in GISTs was observed between the results of Western blotting/real-time PCR and that of immunohistochemistry. Western blotting and real-time PCR revealed that almost all small intestinal GISTs had high *CADM1* expression, while immunohistochemistry showed that considerable cases of small intestinal GISTs were not apparently positive for CADM1. The antibody used might not be good enough to detect the CADM1 signal by immunohistochemistry.

As described above, expression of CADM1 might be associated with invasiveness of leukemic cells [32, 33]. Moreover, CADM1 expression in malignant pleural mesothelioma appeared to be associated with efficient adhesion and growth on CADM1 expressing mesothelium [34]. Furthermore, it has been recently reported that CADM1 might play an important role on peritoneal dissemination of tumor cells of signet ring cell carcinoma through promoting their adhesion to peritoneal cells and their growth in the serosal tissue [35]. High expression of *CADM1* in small intestinal GISTs possibly resulting in promoting tumor invasion and tumor growth on the peritoneum (peritoneal dissemination) might explain the more aggressive behavior and worse prognosis of small intestinal GISTs. We are now planning to examine the role of CADM1 on invasiveness and metastasis of cultured GIST cells using *in vitro* and *in vivo* experiments. The functional experiments might clarify whether CADM1 is truly associated with worse prognosis in patients with small intestinal GISTs. Moreover, there is a possibility that targeting therapy against CADM1 has a preventive effect for vessel infiltration and peritoneal dissemination in GISTs.

## Data Availability

The authors acknowledge that the data presented in this study must be deposited and made publicly available in an acceptable repository, prior to publication. Frontiers cannot accept a article that does not adhere to our open data policies.
