# Supplementary material for: Differential Expression of CADM1 in Gastrointestinal Stromal Tumors of Different Sites and with Different Gene Abnormalities
Source: Pathol Oncol Res. 2021 Apr 19;27:602008. doi: 10.3389/pore.2021.602008 (PMC8262239; doi:10.3389/pore.2021.602008)
Supplement: Supplementary file 1 [file Table1.DOCX]

**Supplementary Table 1** List of primer sequences used for *CADM1* cDNA sequencing in this study

Forward primers

5’-GACATGGCGAGTGTAGTGCT-3’

5’-GCGGTGGGAAGGTGAGGAGAT-3’

Reverse primers

5’-CTCGCAAGTTCCAATATCAC-3’

5’-ACCCCATCGTCCTCCTTGTG-3’
